# Die Generationen Y und Z – Neue Herausforderungen für Führungskräfte im Krankenhaus

**DOI:** 10.1007/s00101-021-01026-2

**Published:** 2021-12-02

**Authors:** Reiner M. Waeschle, Christian Schmidt, Antje-Britta Mörstedt

**Affiliations:** 1 Klinik für Anästhesiologie, Universitätsmedizin Göttingen, Robert-Koch-Str. 40, 37099 Göttingen, Deutschland; 2 Vorstand, Universitätsmedizin Rostock, Rostock, Deutschland; 3 Präsidium, PFH, Private University of Applied Science, Göttingen, Deutschland

**Keywords:** Generation, Mitarbeiterführung, Personalentwicklung, Arbeitsmarkt, Berufseinsteiger, Generation, Employee management, Personnel development, Labor market, Young professionals

## Abstract

**Hintergrund und Ziel der Arbeit:**

In dieser Studie wurden die Studierenden der Generationen Y und Z des Studiengangs Humanmedizin an der Georg-August-Universität Göttingen hinsichtlich ihrer Präferenzen am Arbeitsplatz und der Wahl eines Arbeitgebers befragt.

**Material und Methoden:**

Die Befragung wurde 2016 über einen Onlinefragebogen über EvaSys an 2637 Studierende der Universitätsmedizin Göttingen versandt und wurde von 742 beantwortet (Rücklaufquote: 28,1 %).

**Ergebnisse:**

Die Befragten fühlten sich unabhängig von der Generationszugehörigkeit mit 89,5 % überwiegend „gut“ und „nicht so gut“ auf den Übergang von der Universität ins Berufsleben vorbereitet. Die häufigsten Ängste waren viel Arbeit/wenig Freizeit, Zeitmangel bei der Patientenversorgung, mangelnde Erfahrung, Stress, fachliche Überforderung und schlechte Einarbeitung. Die Beschaffung von Informationen über zukünftige Arbeitgeber erfolgte in beiden Gruppen überwiegend mit knapp 60 % über die Homepage des Krankenhauses. Wichtig waren den Studierenden eine besonders gute Ausbildung und eine strukturierte Einarbeitung/ein strukturiertes Weiterbildungscurriculum.

**Diskussion:**

Die Weiterentwicklung eines strukturierten Einarbeitungskonzepts für Berufseinsteiger, ein verbindliches, strukturiertes Weiterbildungscurriculum, die Etablierung bzw. Weiterentwicklung eines Mentoring-Programms, die Weiterentwicklung von Führungsqualitäten und die Gestaltung einer ansprechenden und aktualisierten Homepage sind wesentliche Voraussetzungen, um auf dem hart umkämpften Fachkräftemarkt zukünftig erfolgreich zu sein.

## Abstract (en)

**Background and objective:**

In this study medical students of the generations Y and Z at the Georg-August University Göttingen were asked about their preferences in the workplace and the choice of an employer.

**Material and methods:**

In 2016, the survey was sent to 2637 students at the University Medical Center Göttingen using an online questionnaire via EvaSys and was answered by 742 (response rate: 28.1%).

**Results:**

Regardless of the generation, with 89.5% the respondents felt predominantly “well” and “not so well” prepared for the transition from university to professional life. The most common fears were a lot of work/little free time, lack of time in patient care, lack of experience, stress, excessive technical demands and poor familiarization. In both groups almost 60% obtained information about future employers via the hospital’s website. It was important for the students to have a particularly good education and a structured induction/advanced training curriculum.

**Conclusion:**

The further development of a structured induction concept for young professionals, a binding and structured further training curriculum, the establishment or further development of a mentoring program, the further development of leadership skills and the design of an appealing and updated website are essential prerequisites for future success in the highly competitive skilled labor market.

## Hintergrund und Fragestellung

Der Fachkräftemangel an deutschen Krankenhäusern ist allgegenwärtig und nimmt weiter zu. Er stellt die Krankenhäuser vor neue Herausforderungen hinsichtlich Mitarbeiterakquise, -führung und -entwicklung [1–3]. Wesentliche Aspekte eines solch vielschichtigen Mitarbeitermanagements ist dabei die Berücksichtigung unterschiedlicher Ansprüche und Prioritäten der verschiedenen Generationen. Das Thema „Generationenunterschiede in der Arbeitswelt“ wurde in den vergangenen Jahren auch in zahlreichen Zeitungsartikeln und wissenschaftlichen Publikationen behandelt [4–8]. Dabei wurden in unterschiedlichen Studien vorrangig Vertreter der Generation Y (Jahrgänge von 1980 bis 1994) im Hinblick auf ihre Präferenzen an den Arbeitsplatz befragt [9]. Bisher sind jedoch keine Untersuchungen zur Generation Z durchgeführt worden.

Diese Generation Z beschreibt die Geburtsjahrgänge ab 1994 – also aktuell 27 Jahre und jünger. Sie wird als realistisch, sicherheitsorientiert und anspruchsvoll bezeichnet. Die Vertreter der Generation Z wurden von ihren Eltern umfassend umsorgt und weitgehend aus der Verantwortung für eigene Leistungen genommen [10, 11]. Die Verbreitung des Internets ab 1995 wird für diese Gruppe als wegweisendes Einflusskriterium gesehen, da es die ersten Generation ist, die bereits von Geburt an mit elektronischen Medien wie Smartphone und Touchpads aufwächst [12, 13]. Durch die starke Durchdringung mobiler Kommunikationsmittel in dieser Generation haben sich auch die damit verbundenen Kommunikationsplattformen geändert. Während sich Generation Y noch über Facebook und Twitter ausgetauscht hat, sind bei Generation Z Snapchat, TikTok und Telegram hinzugekommen. Die McDonalds-Studie 2017 zeigt darüber hinaus, dass Spaß im Berufsleben und eine kollegiale Arbeitsatmosphäre weitere wichtige Erwartungen an den Arbeitsplatz sind [14].

Im Rahmen dieser prospektiven Umfrage unter den Medizinstudenten der Generation Y und Z der Georg-August-Universität Göttingen sollten Veränderungen hinsichtlich Kommunikationskultur und Anforderungen an Vorgesetzte sowie weitere Aspekte hinsichtlich Präferenzen am Arbeitsplatz und der Wahl eines Arbeitgebers objektiviert werden.

## Studiendesign und Untersuchungsmethoden

### Fragebogen

Der Fragebogen umfasste 47 Fragen zu folgenden Aspekten:

- Fragen zur Person,
- Fragen zum Medizinstudium und zum Übergang in das Berufsleben,
- Fragen zu Erwartungen an einen zukünftigen Arbeitgeber (wie Ausbildung, Führungsqualitäten, Finanzielles, Arbeitsbedingungen),
- Fragen zur Arbeitsplatzsuche und zu Erwartungen hinsichtlich der IT-Infrastruktur am Arbeitsplatz,
- Fragen zur Nutzung neuer Technologien im Berufsleben.

Die Fragetypen bestanden aus dichotomen Fragen, Fragen mit 4‑stufiger Ordinalskala und Fragen mit Mehrfachantworten. Der erforderliche Zeitaufwand für die Bearbeitung betrug 10 min.

### Umfrage

Die anonyme Umfrage wurde mittels der Onlineplattform EvaSys Version 7.1 (evasys GmbH, Lüneburg, Deutschland) zum 23.05.2016 gestartet. Der E‑Mail-Verteiler umfasste 2637 Medizinstudenten der Universitätsmedizin Göttingen. Jede der versandten E‑Mails enthielt einen Link zur Beantwortung eines einzelnen, online bereitgestellten Fragebogens. Es wurden 3 automatisierte, elektronische Erinnerung an die Befragten aus dem Verteiler versandt, die noch nicht teilgenommen hatten. Die Datenerhebung wurde am 24.10.2016 abgeschlossen. Bis zu diesem Zeitpunkt waren 742 Fragebogen beantwortet (Rücklaufquote: 28,1 %).

### Datenauswertung

Von den angeschriebenen Studierenden haben 742 den Fragebogen beantwortet (Rücklaufquote: 28,1 %). Aufgrund der Altersverteilung und der Zugangsformen zum Humanmedizinstudium an der Georg-August-Universität Göttingen ist von einer vergleichbaren Rücklaufquote für die Generationen Z und Y auszugehen.

Aufgrund der beschriebenen Fragestellung wurden folgende Generationen unterschieden:

- Generation Y: Geburtsjahrgang 1980–1993 [15],
- Generation Z: Geburtsjahrgang 1994–2010 [15].

Von den 742 antwortenden Studierenden wurden 11 Fragebogen ausgeschlossen, da kein Geburtsjahr angegeben war, und weitere 6 Fragebogen wurden durch Studierende der Generation X (vor 1980) ausgefüllt und damit exkludiert.

Die Anzahl der berücksichtigten 725 Antwortenden der beiden Generationen verteilt sich wie folgt:

- Generation Z: 145 Antwortende,
- Generation Y: 580 Antwortende.

Die weitere Auswertung der Daten erfolgte unter Verwendung des Datenbankprogramms Access 2010 (Microsoft© Corporation, Redmond, WA, USA) und der Business Intelligence Software QlikView (Version 11, Qlik Tech©, Radnor, PA, USA).

### Deskriptive Statistik

Da bei verschiedenen Fragen Mehrfachantworten zugelassen waren, wurden innerhalb jeder Generationskategorien die Anzahl der gegebenen Antworten auf die Anzahl beantworteter Fragebogen bezogen. Diese Herangehensweise führte dazu, dass bei Fragen mit Mehrfachantworten eine kumulative Prozentzahl über 100 % innerhalb jeder Generation möglich ist.

Die statistische Auswertung erfolgte mittels nichtparametrischen Verfahren. Dabei wurde in Abhängigkeit von der Variablenkonstellation 2 × 2-Felder-Tafeln bzw. der Pearson-Chi-Quadrat-Test verwendet. Das Signifikanzniveau wurde <0,05 definiert.

## Ergebnisse

### Allgemeine Fragen

Eine Übersicht der Ergebnisse ist in Tab. 1 dargestellt.

**Table 1:** 

	Generation Z	Generation Y	*p*-Wert
**Allgemeine Fragen**			
Geschlecht, weiblich	68,3 %/99	62,6 %/363	0,3689
Geburtsjahr (Mittelwert/Standardabweichung)	1995 (± 1,0)	1989 (± 2,8)	–
Studienabschnitt			
– Vorklinik	73,8 %/107	15,3 %/89	**<0,0001**
– Klinik	25,5 %/37	60,3 %/350	
– Praktisches Jahr	0,0 %/0	21,6 %/125	
Familienstand			
– Single	66,2 %/96	40,9 %/237	**<0,0001**
– In einer Beziehung	33,8 %/49	50,9 %/295	
– Verheiratet	–	7,9 %/46	
– Geschieden	–	0,2 %/1	
Kinder bekommen: „Ja“	0,7 %/1	6,6 %/38	
*Nach dem Abitur*			
– Medizinstudium	34,7 %/50	21,8 %/126	**<0,0001**
– Freiwilliges soziales/ökologisches Jahr	18,1 %/26	9,3 %/54	
– Auslandsaufenthalt	15,3 %/22	11,1 %/64	
– Ausbildung im Gesundheitswesen (MTA, Arzthelfer etc.)	11,1 %/16	17,6 %/102	
– Anderer Studiengang	4,9 %/7	11,4 %/66	
– Pflegerische Ausbildung	1,4 %/2	14,3 %/83	
– Sonstiges	14,6 %/21 (Praktika, Ausbildung, Arbeit)	14,5 %/84 (Zivildienst, Ausbildung, Arbeit)	
*Nutzung sozialer Medien*			
– WhatsApp	94,5 %/137	89,1 %/517	**<0,0001**
– Facebook	86,9 %/126	82,8 %/480	
– YouTube	74,5 %/108	62,4 %/362	
– Instagram	24,1 %/35	13,4 %/78	
– Snapchat	24,1 %/35	7,2 %/42	
– Tumblr	3,4 %/5	0,9 %/5	
– Twitter	2,8 %/4	4,0 %/23	
*Beweggrund für das Medizinstudium*			
– Interesse an Gesundheit und Pflege	90,3 %/131	89,7 %/520	0,2555
– Wunsch, Menschen zu helfen	69,0 %/100	54,1 %/314	
– Gute Verdienstmöglichkeiten	23,4 %/34	29,3 %/170	
– Andere Studiengänge sind weniger interessant	21,4 %/31	25,2 %/146	
– Verwandte arbeiten im Gesundheitswesen arbeiten	21,4 %/31	21,0 %/122	
*Einschätzung der Chancen auf dem Arbeitsmarkt*			
– Sehr optimistisch	51,0 %/74	58,1 %/336	0,1966
– Eher optimistisch	47,6 %/69	39,4 %/228	
– Eher pessimistisch	1,4 %/2	1,2 %/7	
– Sehr pessimistisch	0,0 %/0	0,0 %/0	
– Weiß nicht	0,0 %/0	1,2 %/7	
**Medizinstudium und Berufseinstieg**			
*Vorbereitung auf den Übergang von der Universität ins Berufsleben*			
– Sehr gut	2,1 %/3	1,7 %/10	*p* = 0,9688
– Gut	37,9 %/55	37,9 %/220	
– Nicht so gut	51,7 %/75	51,6 %/299	
– Gar nicht	2,1 %/3	1,6 %/9	
*Quellen, Informationsbeschaffung zur Klärung fachlicher Fragen*			
– Bücher/Bibliotheken	90,3 %/131	82,6 %/479	***p*** **<** **0,0001**
– Kommilitonen	78,6 %/114	63,4 %/368	
– Online-Fachinformationen (*DocCheck, Amboss, Thieme-Online* etc.)	69,7 %/101	76,2 %/442	
– Suchmaschinen	60,7 %/88	55,2 %/320	
– Dozenten	23,4 %/34	37,6 %/218	
– Wikipedia	17,9 %/26	29,0 %/168	
– Semester-Kurznachrichtengruppe	15,2 %/22	7,9 %/46	
– Eltern	11,0 %/16	4,8 %/28	
– Youtube	6,9 %/10	7,9 %/46	
– Vorgesetzte	0,0 %/0	5,3 %/31	
*Wo wollen die Studierenden später arbeiten?*			
– Selbstständige Arbeit in einer eigenen Praxis bzw. einer Gemeinschaftspraxis	42,8 %/62	26,4 %/153	***p*** **=** **0,0007**
– Universitätsklinikum	42,8 %/62	25,2 %/146	
– Krankenhaus der Maximalversorgung	16,6 %/24	27,1 %/157	
– Krankenhaus der Schwerpunktversorgung	5,5 %/8	3,6 %/21	
– Krankenhaus der Grund- und Regelversorgung	4,8 %/7	10,9 %/63	
– Krankenhaus in privater Trägerschaft	3,4 %/5	1,6 %/9	
– Medizinische Versorgungszentrum	3,4 %/5	4,0 %/23	
*Wo die Studierenden sich explizit nicht vorstellen können zu arbeiten*			
– Krankenhaus der Grund- und Regelversorgung	15,9 %/23	15,5 %/90	0,1965
– Universitätsklinikum	11,7 %/17	26,6 %/154	
– Krankenhaus in privater Trägerschaft	8,3 %/12	12,6 %/73	
– Krankenhaus der Maximalversorgung	7,6 %/11	6,7 %/39	
– Selbstständige Arbeit in einer eigenen Praxis bzw. einer Gemeinschaftspraxis	6,9 %/10	9,8 %/57	
– Medizinische Versorgungszentrum	6,2 %/9	10,2 %/59	
– Krankenhaus der Schwerpunktversorgung	3,4 %/5	6,6 %/38	
*In welchem Fachbereich die Studierenden später arbeiten wollen*			
– Innere Medizin	35,2 %/51	47,4 %/275	**0,0031**
– Allgemeinmedizin	32,4 %/47	29,1 %/169	
– Pädiatrie	29,0 %/42	21,6 %/125	
– Unfallchirurgie/Orthopädie	20,7 %/30	14,8 %/86	
– Neurologie	20,0 %/29	14,3 %/83	
– Anästhesiologie	16,6 %/24	28,6 %/166	
– Allgemeinchirurgie	15,2 %/22	12,8 %/74	
– Gynäkologie und Geburtshilfe	15,2 %/22	10,2 %/59	
– Psychiatrie	13,1 %/19	10,3 %/60	
– Plastische/Rekonstruktive Chirurgie	10,3 %/15	7,2 %/42	
– Herz-Thorax-Gefäßchirurgie	9,7 %/14	5,7 %/33	
– Radiologie	8,3 %/12	11,9 %/69	
– Neurochirurgie	6,9 %/10	3,4 %/20	
– Klinische Forschung	6,2 %/9	4,1 %/24	
– Hals-Nasen-Ohren-Heilkunde	4,8 %/7	6,6 %/38	
– Mikrobiologie/Tropenmedizin	4,1 %/6	3,1 %/18	
– Augenheilkunde	3,4 %/5	5,2 %/30	
– Mund-Kiefer-Gesichtschirurgie	3,4 %/5	1,7 %/10	
– Urologie	3,4 %/5	5,5 %/32	
– Labormedizin	3,4 %/5	1,4 %/8	
– Pathologie	2,8 %/4	1,7 %/10	
– Dermatologie	2,1 %/3	7,6 %/44	
– Krankenhaushygiene	0,0 %/0	0,5 %/3	
*Größe der Stadt*			
– Kleine Großstadt (100.000–200.000 z. B. Göttingen, Heidelberg)	35,9 %/52	37,8 %/219	0,5673
– Mittlere Großstadt (200.001–500.000 z. B. Freiburg, Kiel)	26,9 %/39	26,7 %/155	
– Ländliche Region (Kleiner 100.000 Einwohner)	20,7 %/30	16,2 %/94	
– Großstadt (Ab 500.001 – z. B. Köln, Hamburg)	15,9 %/23	18,8 %/109	
*Informationsquellen, zukünftige Arbeitgeber*			
– Firmenwebsite (Stellenausschreibung, Karrierebereich)	57,9 %/84	61,0 %/354	0,1366
– Deutsches Ärzteblatt	31,0 %/45	33,6 %/195	
– Online-Jobbörse (*Stepstone/Monster* o. Ä.)	28,3 %/41	26,4 %/153	
– Dozenten/Vorgesetzte	26,2 %/38	29,5 %/171	
– Soziale Medien (*Facebook, Instagram, YouTube* etc.)	23,4 %/34	25,5 %/148	
– Karrierenetzwerke (*Xing, LinkedIn* etc.)	15,9 %/23	17,4 %/101	
– Berufsmessen	15,9 %/23	14,8 %/86	
– Eltern	15,9 %/23	7,1 %/41	
– Tageszeitungen	11,7 %/17	9,1 %/53	

*FSJ* Freiwilliges soziales Jahr

### Medizinstudium und Berufseinstieg

Eine Auswahl der Umfrageergebnisse findet sich ebenfalls in Tab. 1.

**Figure 1:**
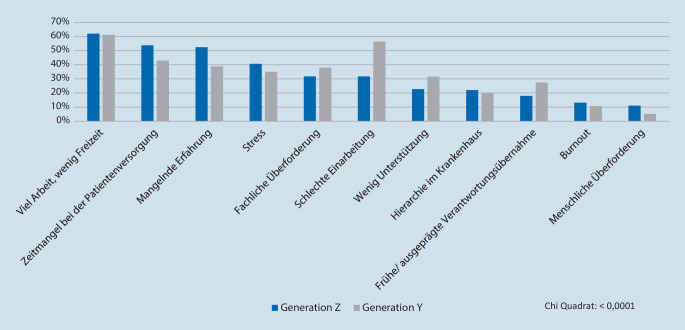

Die Ängste der Studierenden hinsichtlich des Einstiegs in das ärztliche Berufsleben zeigen signifikante Unterschiede (*p* < 0,0001) und sind in Abb. 1 dargestellt.

### Erwartungen an einen zukünftigen Arbeitgeber

**Figure 2:**
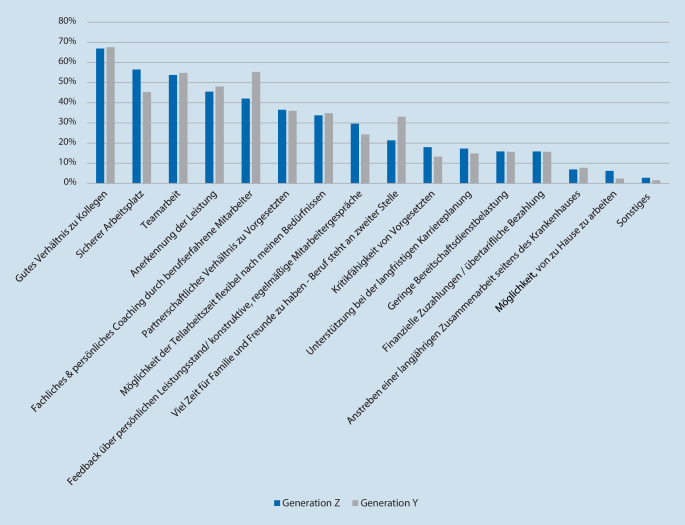

Bei der Frage, was den Student*innen im Berufsleben wichtig ist, zeigen sich keine signifikanten Unterschiede (*p* = 0,1016). Die Ergebnisse sind in Abb. 2 dargestellt.

**Figure 3:**
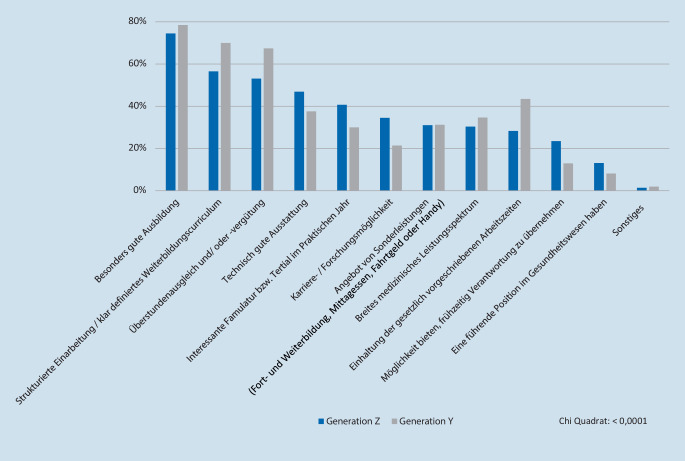

Die Antworten auf die Frage „Was ist Ihnen bei einem potenziellen Arbeitgeber wichtig?“ waren signifikant unterschiedlich (*p* < 0,0001) und sind in Abb. 3 gezeigt.

Bei den Sonderleistungen wurde die Finanzierung von Fort- und Weiterbildung überwiegend gewählt (Gen Z: 35/85,4 % vs. Gen Y: 140/85,9 %).

Die Aufgabe, die Begriffe „Arbeitsbedingungen“, „Freizeit/Familie“, „Ausbildung“, „Karriere“, „finanzielle Aspekte“ und „Arbeitsvertrag“ einer Priorität zuzuordnen, führte zu einer ähnlichen Verteilung in beiden Gruppen. So wurden die Arbeitsbedingungen als erste Priorität am häufigsten genannt (Gen. Z: 42,8 %/62 vs. Gen. Y: 47,8 %/277). An zweiter und dritter Stelle kamen die Antwortmöglichkeiten „Freizeit/Familie“ (Gen. Z: 37,2 %/54 vs. Gen. Y: 32,1 %/186) und „Ausbildung“ (Gen. Z: 31,0 %/45 vs. Gen. Y: 27,8 %/161). Die vierte Priorität war in beiden Gruppen „finanzielle Aspekte“ (Gen. Z: 30,3 %/44 vs. Gen. Y: 30,9 %/179). Die letzten beiden Prioritäten waren unterschiedlich besetzt. So wählte die Generation Z an 5. Stelle ebenfalls am häufigsten „finanzielle Aspekte“ (33,1 %/48) und an letzter Stelle den „Arbeitsvertrag“ (37,9 %/55). Die Generation Y entschied sich für den „Arbeitsvertrag“ an 5. Stelle (31,0 %/180) und die Karriere als letzte Priorität (48,4 %/281).

Die Frage nach Einsatzmöglichkeiten sozialer Medien bzw. deren bisherigen Nutzung im Arbeitsumfeld umfasste die Antwortmöglichkeiten „Messenger-Dienste zur Abstimmung mit Kolleg*innen“, „Intranet als App“, „Online-Webinare“, „eLearning“, „Facebook im Krankenhaus“ und „Ich kann mir soziale Medien im Arbeitsumfeld nicht als nützliche Ergänzung vorstellen“ und zeigte keine signifikanten Unterschiede (*p* = 0,0717). Die Generation Z gab am häufigsten die „Messenger-Dienste zur Abstimmung mit Kolleg*innen“ (Gen. Z: 63,4 %/92 vs. Gen. Y: 54,1 %/314) und das „Intranet als App“ (Gen. Z: 41,4 %/60 vs. Gen. Y: 47,8 %/277) als nützliche Anwendungen an. Nachfolgend waren „Online-Webinare“ (Gen. Z: 40,0 %/58 vs. Gen. Y: 42,1 %/244) und „eLearning“ (Gen. Z: 35,9 %/52 vs. Gen. Y: 52,9 %/307) am häufigsten vertreten, wobei Letzteres am zweithäufigsten durch die Generation Y gewählt worden war. „Facebook im Krankenhaus“ konnten sich beide Generationen nur selten im Arbeitsumfeld vorstellen (Gen. Z: 2,8 %/4 vs. Gen. Y: 1,7 %/10). Die Aussage „Ich kann mir soziale Medien im Arbeitsumfeld nicht als nützliche Ergänzung vorstellen“ wurde in beiden Gruppen durch über 10 % der Befragten bejaht (Gen. Z: 12,4 %/18 vs. Gen. Y: 13,4 %/78).

Die Bewertung verschiedener Einzelaussagen wird in Tab. 2 dargestellt.

**Table 2:** 

	Generation Z				Generation Y				
	Stimme voll zu (%)	Stimme eher zu (%)	Stimme eher nicht zu (%)	Stimme gar nicht zu(%)	Stimme voll zu (%)	Stimme eher zu (%)	Stimme eher nicht zu (%)	Stimme gar nicht zu(%)	*p*-Wert
„Für ein besseres finanzielles Angebot würde ich meinen Arbeitgeber wechseln.“	11,3	66,9	20,4	1,4	16,1	58,7	23,9	1,4	0,3049
„Für ein besseres finanzielles Angebot würde ich meinen Wohnort wechseln“	4,2	38,7	50,0	7,0	5,2	35,6	50,2	9,0	0,7966
„Für eine attraktivere Aufgabe würde ich den Arbeitgeber wechseln“	47,2	51,4	0,7	0,7	42,6	54,1	2,8	0,5	0,4194
„Für eine attraktivere Aufgabe würde ich den Wohnort wechseln“	18,3	58,5	20,4	1,4	20,8	54,2	21,8	2,9	0,6074
**„Freizeit ist für mich wichtiger als mein Job“**	**5,6**	**36,6**	**54,9**	**2,8**	**19,1**	**44,0**	**35,0**	**1,9**	<0,0001*
„Während meiner Ausbildung wünsche ich mir einen Ansprechpartner, der mir bei Problemen und Fragen beratend zur Seite steht“	80,3	17,6	1,4	0,0	81,8	17,2	1,0	0,0	0,9140
„Ich wünsche mir feste und geregelte Arbeitszeiten“	48,6	43,7	7,7	0,0	57,2	35,5	6,4	0,7	0,1845
**„Meine Kollegen und mein Chef können mich bezüglich beruflicher Angelegenheiten gerne auch nach Feierabend anrufen“**	**9,2**	**40,1**	**44,4**	**6,3**	**4,7**	**27,6**	**49,0**	**18,9**	<0,0001*
**„Eine berufliche Karriere ist mir wichtiger als viel Freizeit“**	**7,7**	**24,6**	**54,9**	**11,3**	**4,0**	**18,7**	**50,3**	**26,5**	0,0006*
„Der Social-Media-Auftritt eines Krankenhauses ist für mich ein entscheidender Faktor, ob ich mich bei diesem bewerbe“	2,8	19,7	43,0	33,8	2,1	15,9	40,2	41,1	0,3943
„Mein Chef soll ein Vorbild für mich sein“	64,1	31,7	3,5	0,0	57,4	39,0	3,3	0,2	0,4293
**„Um genügend Zeit für mich zu haben, würde ich gerne weniger als 5 Tage pro Woche arbeiten“**	**1,4**	**14,1**	**59,9**	**24,6**	**12,0**	**25,0**	**45,8**	**17,3**	<0,0001*
**„Ich wünsche mir ein regelmäßiges Feedback durch meinen Vorgesetzten“**	**28,2**	**61,3**	**9,9**	**0,7**	**40,9**	**52,9**	**6,2**	**0,0**	0,0059*
**„Eine informative Karriere-Website des Klinikums ist für mich ein entscheidender Faktor für eine Bewerbung“**	**10,6**	**47,2**	**40,1**	**2,1**	**7,6**	**37,1**	**36,7**	**18,2**	<0,0001*
„Eine strikte Trennung von Arbeits- und Privatleben ist mir wichtig“	22,5	51,4	23,2	2,8	22,4	49,7	25,1	2,9	0,9716
„Eigenverantwortlich und selbstständig zu arbeiten, ist für mich wichtig“	33,1	57,0	9,2	0,0	36,4	56,2	7,3	0,2	0,7694
**„Ein sicheres und unbefristetes Arbeitsverhältnis ist mir sehr wichtig“**	**55,6**	**38,0**	**6,3**	**0,0**	**42,5**	**47,5**	**9,2**	**0,9**	0,0303*
**„Die Möglichkeit, einen Teil meiner Arbeit (z.** **B. Arztbriefe schreiben) von zu Hause aus zu erledigen (Stichwort ‚Homeoffice‘), ist mir wichtig“**	**15,5**	**44,4**	**34,5**	**5,6**	**10,2**	**36,0**	**38,0**	**15,6**	0,0036*
„Eine moderne, digitalisierte Arbeitswelt (Tablet, Smartphone, PC anstatt Stift & Papier) ist mir wichtig“	15,5	33,1	41,5	9,9	15,1	40,7	34,0	10,1	0,3143
„Bezahlte Fortbildungen (u. a. Zusatzqualifikationen wie Akupunktur etc.) nach der Facharztausbildung sind mir wichtig“	49,3	40,1	10,6	0,0	41,9	49,6	8,0	0,3	0,1769
„Flexibel Urlaub nehmen zu können (und nicht ein Jahr im Voraus festlegen zu müssen) ist mir wichtig“	51,4	35,9	10,6	0,7	50,6	38,3	10,7	0,2	0,7204
„Die Möglichkeit, während der Arbeitszeit das Internet privat zu nutzen (z. B. *Facebook, Whatsapp* etc.), ist mir wichtig“	7,7	21,1	44,4	26,8	7,6	23,7	43,0	25,8	0,9346

*<0,05

## Diskussion

Die vorliegende prospektive Studie befasst sich als Erste mit den Themen der Generationen Y und Z im Hinblick auf ihre Präferenzen an den Arbeitsplatz im Krankenhaus und an die Wahl eines Arbeitgebers.

### Allgemeine Fragen und Rücklaufquote

Die Rate der weiblichen Teilnehmer war in beiden Gruppen gleich und betrug im Mittel 68,3 % (Gen Z) bzw. 62,6 % (Gen Y). Diese Rate entspricht dem bundesweiten weiblichen Anteil an Medizinstudenten (im Mittel 61 %) [16]. Die Rücklaufquote lag im Mittel bei 28,1 %, was nach einer Studie des Online-Umfrageprogramms SoSci Survey gut ist, denn dort wird ein Mittelwert von ca. 20 % angegeben [17]. Somit stützen sich die Ergebnisse dieser Umfrage auf eine eher überdurchschnittliche Rücklaufquote und bei über 700 Teilnehmern auf eine umfangreiche Datensammlung.

Die Befragten beider Generationen haben am häufigsten direkt mit dem Medizinstudium begonnen, wobei bei der Generation Z mit 37,7 % ein höherer Anteil als bei der Generation Y mit 21,8 % vorlag. Bei den Befragten, die nicht direkt mit dem Studium angefangen haben, fällt auf, dass die Generation Z mehrheitlich ein freiwilliges soziales/ökologisches Jahr oder einen Auslandsaufenthalt davor absolviert hat. Generation Y hat dagegen eher eine Ausbildung im Gesundheitswesen oder eine pflegerische Ausbildung absolviert. War es bei Generation Y noch die Überbrückung von Wartezeiten auf den Studienplatz, sind es heute eher soziale und sinnstiftende Themen, welche die Generation Z bewegen. Zu diesem Schluss kommt auch eine aktuelle Studie des Workplace Survey von Half [18]: Junge Arbeitnehmer stellen höhere Erwartungen an die Ziele und Mission eines Unternehmens sowie an die Sinnhaftigkeit ihrer Tätigkeit.

Die am häufigsten benutzten sozialen Medien waren für beide Gruppen die Text- und Bild-Messenger-Anwendungen *WhatsApp, Facebook* und die Videoplattform *YouTube*. Auffällig war jedoch, dass Generation Z im Vergleich zu Generation Y die sozialen Medien deutlich stärker nutzt. Dies lässt sich auch dadurch erklären, dass die Generation Z bereits von Geburt an mit elektronischen Medien aufgewachsen ist [13].

Die überwiegend optimistische Wahrnehmung der Chancen auf dem Arbeitsmarkt korreliert mit dem tatsächlichen Bedarf an Ärzt*innen in Deutschland. Diese Wahrnehmung kommt dem Sicherheitsbedürfnis gerade der Generation Z entgegen und macht den Arztberuf für sie gerade im Vergleich zur früheren Stellensituation attraktiver.

### Medizinstudium und Berufseinstieg

Die Vorbereitung auf das Berufsleben wird generationsübergreifend mehrheitlich mit „nicht so gut“ bewertet. Die Ursachen dafür sind sicherlich multifaktoriell und eine Kombination aus intrinsischen (z. B. die eigene Erwartungshaltung) und extrinsischen Faktoren wie z. B. ein eher geringer Praxisanteil im Studium. Hier ist aufgrund des hohen Sicherheitsbedürfnisses der Generation Z empfehlenswert, entsprechende Einarbeitungsangebote, Mentoring-Programme und Coachings anzubieten. Die Umsetzung solcher Maßnahmen stellt auch aufgrund der mangelnden Berücksichtigung von Einarbeitung, Mentoring und Coaching im Krankenhausentgeltsystem eine besondere Herausforderung dar.

Beide Generationen haben die Sorge, dass sie im Berufsleben mit viel Arbeit und wenig Freizeit umgehen müssen. Ebenso gehören der Zeitmangel bei der Patientenversorgung sowie eine mangelnde Erfahrung zu den zentralen Ängsten der Generation Z. Eine schlechte Einarbeitung befürchten vor allem die Befragten der Generation Y. Diese wird auch in anderen Befragungen als besonders wichtig eingeschätzt [19, 20]. Hinsichtlich der Einhaltung von Arbeitszeiten und der Limitierung von Überstunden wurden in den letzten Jahren deutliche Verbesserungen erreicht. Unabhängig davon sind geregelte Arbeitszeiten unverändert für die Befragten überaus wichtig und werden von über 90 % gewünscht. Daher sollten Arbeitgeber darauf achten, dass Dienstplanmodelle diesen Ansprüchen genügen und auch umgesetzt werden. Die vollumfängliche Einhaltung der Regelarbeitszeiten ist in der medizinischen Versorgung jedoch nicht immer möglich und sollte daher bei Abweichungen verständlich erklärt werden.

Beiden Generationen ist ein sicheres und unbefristetes Arbeitsverhältnis wichtig, wobei die Generation Z diese Frage mit 55,6 % voller Zustimmung signifikant eindeutiger beantwortet als die Generation Y mit 42,5 %. Zu diesem Ergebnis kommen ebenfalls weltweite Trendstudien, die den Wunsch der Generation Z nach Sicherheit und geregelten Arbeitszeiten zeigen [21]. Beide Generationen stützen sich bei der Wahl des Arbeitgebers in erster Linie auf die Homepage des Krankenhauses gefolgt vom Deutschen Ärzteblatt und Online Jobbörsen. Diese Ergebnisse verdeutlichen, dass junge Generationen vorrangig über den Internetauftritt der Einrichtung angesprochen werden. Dabei steht nach Klaffke das Wecken von Begeisterung für die jeweiligen Einrichtungen im Vordergrund [12]. Die Homepage muss dem Besucher vermitteln, was diese Klinik als Arbeitgeber interessant macht und warum man sich gerade für diese Klinik entscheiden sollte. Dazu gehören u. a. interessante klinische Tätigkeiten, flexible Arbeitsbedingungen, Informationen zu besonderen Fortbildungsprogrammen, einer strukturierten, verlässlichen Weiterbildung, Mentoring-Programme und besondere klinische Entwicklungsmöglichkeiten. Ein Imagevideo kann dabei eine sinnvolle Ergänzung sein. Um sich als moderner Arbeitgeber zu präsentieren, ist dabei auch die Diversität der Belegschaft und das soziale Engagement zu verdeutlichen [12].

### Erwartungen an einen zukünftigen Arbeitgeber

Bei der Frage nach den Präferenzen am Arbeitsplatz wurden generationsunabhängig das Arbeitsklima, die Sicherheit des Arbeitsplatzes, Teamarbeit, Anerkennung der Leistung und fachliches bzw. persönliches Coaching durch berufserfahrene Mitarbeiter als wichtigste Parameter genannt. Dies zeigt, wie wichtig Führungskompetenz beim Umgang mit diesen Generationen geworden ist, denn einen autoritären Führungsstil lehnen beide Generationen ab. Geschätzt wird dagegen ein partizipativer Führungsstil mit Einbindung der Mitarbeiter in die Entscheidungsfindung im Klinikalltag [4, 18–20]. Dennoch dürfte die Umsetzung dieses Führungsstils nicht immer realisierbar sein, vor allem, wenn Entscheidungen über die Therapie von Ober- oder Chefärzten verantwortet werden müssen. Dennoch sollten die bisweilen ausgeprägt hierarchischen Strukturen vieler Kliniken in diesem Kontext überdacht werden [19, 20, 22]. Hier könnte Generation Z helfen, eine grundsätzliche Veränderung anzustoßen [21].

Den Befragten ist bei einem potenziellen Arbeitgeber eine gute Ausbildung, strukturierte Einarbeitung bzw. klar definiertes Weiterbildungscurriculum und wenige Überstunden wichtig [4, 19, 20]. Darüber hinaus wird auch eine zeitgemäße technische Ausstattung erwartet. Für Forschungsmöglichkeiten interessieren sich 34,5 % (Gen. Z) bzw. 21,4 % (Gen. Y). Um Mitarbeiter für Forschung zu begeistern, muss diese zumindest teilweise innerhalb der Arbeitszeit erbracht werden können. Clinical Scientist Programme sind hier ein sehr guter Ansatz.

Bei möglichen Angeboten von Sonderleistungen interessiert sich der überwiegende Teil beider Generationen für die Finanzierung von Fort- und Weiterbildung durch die Klinik. Hier sollten klare Regelungen im Haus vorhanden sein.

Bei der Priorisierung verschiedener vorgegebener Stichworte wurde die „Arbeitsbedingungen“ an erster Stelle noch vor der „Freizeit/Familie“ und der „Ausbildung“ genannt. Dieses Ergebnis relativiert die beiden Generationen nachgesagte hohe Priorisierung von Freizeit und Familie gegenüber der Arbeit. Folglich ist die Weiterentwicklung der Arbeitsbedingungen ein wichtiger Wettbewerbsfaktor. Dabei ist zu beachten, dass Arbeitsbedingungen als Teil eines generationenübergreifenden Gesamtkonzepts zu verstehen sind, indem die Bedürfnisse aller Mitarbeiter*innen gleichermaßen zu berücksichtigen sind.

Bei der Nutzung sozialer Medien im Arbeitsumfeld werden von diesen Generationen v. a. Messenger-Dienste, Online-Webinare und eLearning-Plattformen favorisiert. Bei den vielfältigen digitalen Kommunikationsmöglichkeiten muss der Datenschutz besonders berücksichtigt werden. Die häufig privat verwendeten Anbieter wie *WhatsApp, Skype* etc. sind für den Austausch sensibler Daten zum jetzigen Zeitpunkt ungeeignet.

Abschließend sei betont, dass die Bedürfnisse der jüngeren Generationen zwar wichtig sind und auch Berücksichtigung finden müssen, allerdings muss dies auch im Kontext der anderen Ärzt*innen geschehen. Diese haben ebenso Wünsche und Bedürfnisse, die nicht in den Hintergrund treten dürfen, weil eine neue Generation nach mehr Aufmerksamkeit verlangt. Generationenkonflikte wären damit vorprogrammiert. Genau hier sind Führungskräfte in Medizin und Pflege gefordert, diese Belange angemessen zu berücksichtigen.

### Limitierungen

Die Befragung und deren Ergebnisse unterliegen Limitationen. Erstens ist der Begriff „Generation“ an sich umstritten und vereinzelt mit der Begründung abgelehnt, dass sämtliche Studien nicht kohortenübergreifend durchgeführt werden [23]. Dieser Kritikpunkt wurde in der vorliegenden Umfrage durch Berücksichtigung beider Generationen relativiert. Es bleibt festzuhalten, dass es eine große Streuung innerhalb von Vertretern einer Generation gibt (Intragenerationsvarianz) und sich damit z. B. Vertreter der Generation Z wie ältere Generationsvertreter verhalten und umgekehrt. Trotz dieser statistischen Streuung gibt es teilweise signifikante Unterscheidungen zwischen den unterschiedlichen Generationen (Intergenerationsdifferenz) [24]. Somit müssen Feststellungen immer kritisch hinterfragt werden.

Es wurden ausschließlich Studierende der Universitätsmedizin in Göttingen befragt. Auch handelt es sich um eine einmalige Befragung. Um nachhaltige Ergebnisse zu erzielen, sollte die Befragung wiederholt werden.

Eine weitere Limitierung besteht darin, dass sich die jüngeren Vertreter der Generation Z eher am Anfang des Studiums und somit in ihrer formativen Phase befinden. Es ist möglich, dass sich Wertevorstellungen und Präferenzen über die Semester mit zunehmender Erfahrung ändern und damit auch Umfrageergebnisse systematisch beeinflusst werden. Dieser Aspekt lässt sich durch wiederholte Befragungen von Medizinstudenten von nachfolgenden Semestern sowie durch wiederholte Befragung desselben Kollektivs im weiteren Studienverlauf bzw. im Berufsleben relativieren. Es ist vorgesehen, diese Umfrage auch in anderen medizinischen Fakultäten durchzuführen, um die Aussagekraft zu erhöhen.

Es bleibt abzuwarten, wie sich z. B. der Ausbruch der COVID-19-Pandemie auf die Generation Z auswirkt. So kommt eine der ersten Studien zu dieser Fragestellung zu dem Ergebnis, dass über 70 % der Befragten in der Generation Y und Z sich neuen Themen zuwenden wollen, um „positiven Einfluss auf ihre Umwelt zu haben“ [25]. Von solchen Entwicklungen können Gesundheitsberufe besonders profitieren.

## Fazit für die Praxis

Basierend auf den in dieser Studie gewonnenen Erkenntnissen leiten sich folgende Empfehlungen ab und können zu einem relevanten Wettbewerbsvorteil bei der Akquise junger Assistenzärzt*innen beitragen:

- Die Weiterentwicklung bzw. Etablierung eines strukturierten Einarbeitungskonzepts für Berufseinsteiger gibt neuen Mitarbeiter*innen Sicherheit und ist eine zentrale Forderung der Generationen Y und Z.
- Ein verbindliches, strukturiertes Weiterbildungscurriculum bietet den jungen Kolleg*innen eine planbare und verlässliche Weiterbildungszeit und damit Sicherheit.
- Die Etablierung bzw. Weiterentwicklung eines Mentoring-Programms nimmt den Berufseinsteiger*innen Ängste und unterstützt einen positiven Start ins ärztliche Berufsleben.
- Die Weiterentwicklung von Führungsqualitäten mit Fokus auf generationenkonformes Führen ist von besonderer Bedeutung, um den Bedürfnissen der unterschiedlichen Generationen nachzukommen und gleichzeitig Generationenkonflikte zu minimieren bzw. zu vermeiden.
- Die Vermeidung von Überstunden ist ein zentrales Anliegen der aktuellen Assistenzärzt*innen und kommenden Berufseinsteiger*innen.
- Die Gestaltung einer ansprechenden und aktualisierten Homepage ist wesentlich für die Akquise neuer ärztlicher Mitarbeiter*innen.
- Die Bereitstellung eines schnellen Internetzugangs für die Mitarbeiter*innen ist für die Vertreter der Generationen Y und Z wesentliche Grundvoraussetzung für die Verwendung von Messenger Diensten und die Erreichbarkeit online verfügbaren medizinischen Fachwissens.
